# Influence of environmental factors in the adherence of an atypical enteropathogenic *Escherichia coli* strain to epithelial cells

**DOI:** 10.1186/s12866-014-0299-y

**Published:** 2014-12-20

**Authors:** Fabiano T Romão, Rodrigo T Hernandes, Denise Yamamoto, Lika Osugui, Ana Flavia Popi, Tânia A T Gomes

**Affiliations:** Departamento de Microbiologia, Imunologia e Parasitologia, Universidade Federal de São Paulo (UNIFESP), Escola Paulista de Medicina, São Paulo, SP Brazil; Departamento de Microbiologia e Imunologia, Instituto de Biociências, Universidade Estadual Paulista (UNESP), Botucatu, SP Brazil

**Keywords:** Atypical EPEC, Glucose, type I pilus, Adherence, Environmental factors, Diarrhea

## Abstract

**Background:**

Attachment is essential to maintain bacteria at their preferential intestinal colonization sites. There is little information on the influence of different environmental conditions in the interaction of atypical enteropathogenic *Escherichia coli* (aEPEC) strains with epithelial cells. In this study, we evaluated the effect of different glucose (5 and 25 mM) and CO_2_ (0.03 and 5%) concentrations and presence of bile salts on the adhesiveness of the aEPEC strain 1551–2.

**Results:**

We found that a CO_2_-enriched atmosphere enhanced the adhesiveness of the aEPEC 1551–2 strain independently of glucose concentrations or presence of bile salts. Conversely, the presence of high glucose concentration altered the original localized adherence (LA) pattern observed at 5 mM glucose, which is characterized by the formation of compact bacterial clusters, to a hybrid adherence pattern (LA and an aggregative adherence-like pattern). In addition, at high glucose concentration, there was increased expression of the *fim*A gene, which encodes the major subunit of type 1 pilus (T1P), and an isogenic *fimA* mutant displayed only LA. The presence of bile salts did not interfere with the adhesion properties of the 1551–2 strain to HeLa cells.

**Conclusions:**

Our data suggest that a CO_2_-enriched atmosphere could favor aEPEC adhesion to the host cells, whereas enhanced T1P production under high glucose concentration could allow bacteria to access more extensive intestinal colonization sites in the host at the beginning of the infectious process.

## Background

Enteropathogenic *Escherichia coli* (EPEC) is an important cause of gastroenteritis in humans [1]. The EPEC pathotype is subdivided into typical EPEC (tEPEC) and atypical EPEC (aEPEC), where the main difference between the two subgroups is the presence of the large virulence EAF (EPEC adherence factor) plasmid (pEAF) in tEPEC and its absence in aEPEC [2,3]. pEAF encodes bundle-forming pilus (BFP), a type IV fimbria, which mediates a localized adherence (LA) pattern *in vitro*, and is characterized by the formation of compact microcolonies on the surface of HeLa/HEp-2 cells [4,5].

The hallmark of EPEC pathogenesis is a histopathologic lesion termed attaching and effacing (AE) lesion, which is characterized by intimate bacterial adherence to enterocytes and effacement of microvilli [6]. The AE lesion is a consequence of the interaction between the outer membrane protein intimin and its receptor Tir (translocated intimin receptor), which is translocated through a type 3 secretion system (T3SS) into eukaryotic cells [7,8]. The genes involved in the AE lesion phenotype are localized in the pathogenicity island termed locus of enterocyte effacement or LEE region [9].

Attachment comprises a critical stage to avoid displacement of bacteria from its preferential site in the gut by the continuous flow of intestinal contents. The initial step in attachment of different diarrheagenic *E. coli* (DEC) pathotypes to the host intestinal epithelium is usually mediated by fimbrial and afimbrial adhesins [10,11]. Previous studies have demonstrated that environmental conditions may regulate the expression of DEC virulence-encoding genes including adhesins and T3SS-dependent effectors [12–15]. In a tEPEC strain, serotype O111:H^−^, Puente and coworkers found that BFP expression was increased at higher glucose concentrations, suggesting that tEPEC preferentially colonizes the proximal small intestine, where the glucose concentration is greater than 25 mM [16]. This hypothesis was further confirmed in experiments employing an *in vitro* organ culture (IVOC) adhesion assay with intestinal tissues obtained from children [17].

Several studies in the literature have focused on identifying new putative adhesive structures, and the adherence pattern resulting from the interaction between these bacterial structures and host cells. However, particularly in aEPEC, very few studies have tried to understand how the intestinal environment may favor or prevent this interaction.

We previously identified an aEPEC strain, 1551–2 (serotype ONT:H^−^), which displays the LA pattern in HeLa cells in the absence of BFP [18]. In the present study we evaluated the adherence pattern and colonization efficiency of aEPEC 1551–2 in HeLa cells cultivated at different CO_2_ and glucose concentrations and in the presence of bile salts.

## Results

### aEPEC 1551–2 strain adheres more efficiently to HeLa cells in an atmosphere supplemented with CO_2_

**Figure 1:**
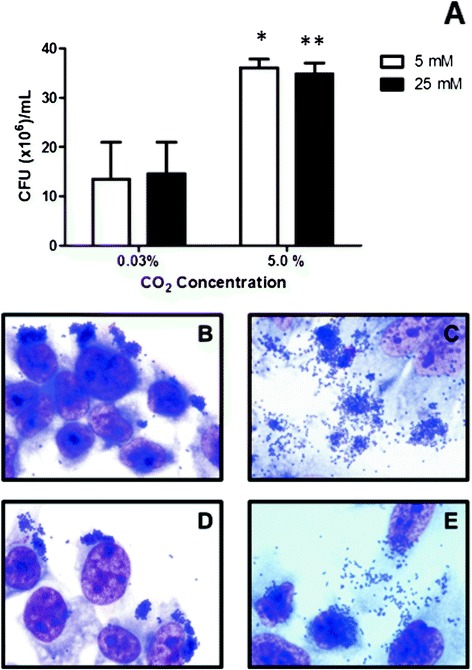
**Comparison of quantitative and qualitative adherence of aEPEC 1551–2 strain to HeLa cells under different environmental conditions. (A)** Number of adherent bacteria recovered after 6 h of incubation with HeLa cells, demonstrating the positive influence of CO_2_ (5%) in the adhesiveness of aEPEC 1551–2. **(B, C, D and E)** Distinct adherence patterns observed in 1551–2 interaction with HeLa cells, after incubation in medium containing 5 mM **(B and D)** or 25 mM **(C and E)** glucose, in a normal air atmosphere **(B and C)** and in an atmosphere of 5% CO_2_ **(D and E)**. The hybrid LA/AA-like pattern is observed over and around cells, only in assays performed in medium containing 25 mM glucose **(C and E)**. * and ** represent statistical differences observed between experiments performed under 0.03% and 5% CO_2_ concentration, in medium containing low (*P* =0.03) or high (*P* =0.002) glucose concentration, respectively.

Quantitative assays demonstrated that the different glucose concentrations tested (5 and 25 mM) did not promote substantial alterations in the adhesiveness of aEPEC 1551–2 (*P* >0.5), as observed in Figure 1A. However, air atmosphere with 5% CO_2_ favored bacterial-cell interaction, since the number of associated bacteria increased 2.3-fold at 5 mM glucose and 1.7-fold at 25 mM glucose, when compared with results obtained in assays performed in a normal air atmosphere (approximately 0.03% CO_2_) (*P* <0.05).

To exclude the possibility of differences in bacterial growth rate in the different environmental conditions tested, a growth curve (measuring the OD_600_) was determined. Bacterial growth rates were not affected by the different conditions tested (data not shown), indicating that the difference observed in the quantitative adherence assays was due to the modification of the air atmosphere by the elevation of CO_2_ concentration to 5%.

### High glucose concentration modifies the adherence pattern of 1551–2 strain to HeLa cells

Regarding the aEPEC 1551–2 adherence pattern, the LA originally identified by Vieira and coworkers [18] was only detected in the presence of low glucose, independently of the CO_2_ concentration used (Figure 1B and D). On the other hand, at high glucose concentration, we identified a hybrid adherence pattern, in which LA was observed concurrently with an aggregative adherence-like (AA-like) pattern on the HeLa cell and on the glass slide surface (abiotic surface) (Figure 1C and E).

### The AA-like pattern of 1551–2 strain is dependent on type 1 pilus (T1P) production at high glucose concentration

**Figure 2:**
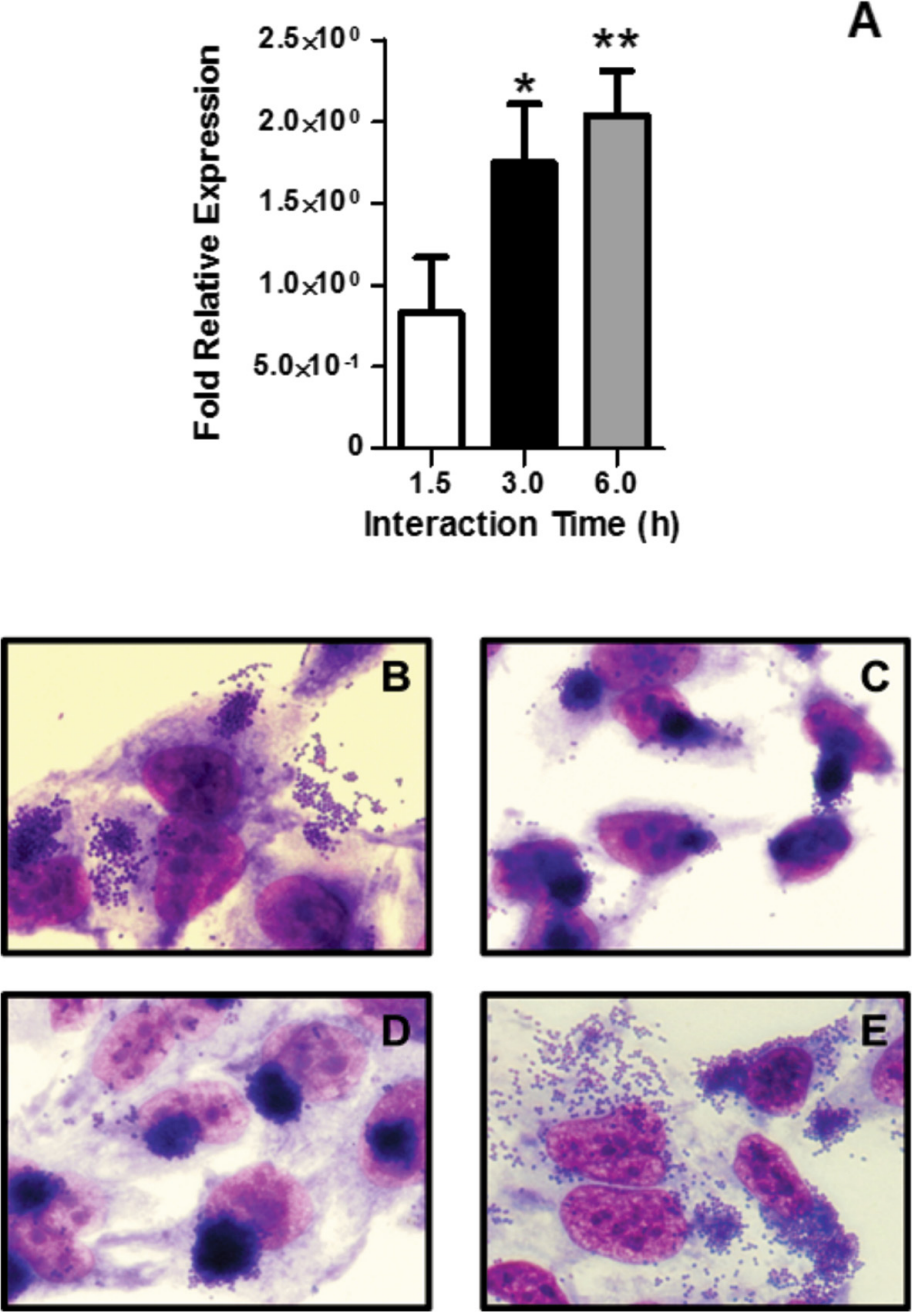
**The aggregative adherence-like pattern shown by aEPEC 1551–2 at high glucose concentration is dependent on type 1 pili production. (A)** Relative expression of *fim*A in presence of 5 mM (calibrator) and 25 mM glucose at 1.5 h, 3 h and 6 h. The data showed that at 3 and 6 h, there was an increase in *fimA* expression of 75 ± 21% and 103 ± 15%, respectively. **(B, C, D and E)** Adherence patterns observed with the wild-type strain **(B)**, 1551-2Δ*fimA* **(C)**, 1551-2Δ*fim*A (pBAD) **(D)** and 1551-2Δ*fim*A (pFimA) **(E)**. The 1551-2Δ*fim*A mutant maintained the LA pattern on HeLa cells, but the AA-like pattern was no longer observed, pointing to the involvement of T1P in the establishment of this phenotype. * and ** indicate statistical differences observed between experiments performed at 5 and 25 mM glucose after 3 h (*P* =0.02) and 6 h (*P* =0.002) of incubation, respectively.

Since T1P is so far the only adhesin, besides intimin, identified in the 1551–2 strain [18–20], we hypothesized that the AA-like pattern would depend on T1P production. To investigate the contribution of T1P in the establishment of the AA-like pattern, we first evaluated the relative expression of the *fim*A gene (encodes for monomer that forms fimbriae) at two glucose concentrations (5 and 25 mM). By comparing the experiments performed in medium supplemented with different glucose concentrations, it was possible to observe an increase in *fim*A gene expression (1.75-fold and 2.03-fold, after 3 and 6 h of incubation, respectively) in experiments performed in medium with high glucose (Figure 2A).

Furthermore, we employed an isogenic *fimA* mutant (1551-2Δ*fimA*) to test the adherence pattern of this strain at high glucose. These experiments demonstrated that in contrast with the wild type aEPEC 1551–2 (Figure 2B), the 1551-2Δ*fimA* strain displayed only the LA pattern, while the AA-like pattern was completely abolished (Figure 2C). These findings clearly point to an association between glucose concentration and T1P production, since the isogenic mutant no longer produced the AA-like pattern, even in the presence of high glucose concentration (Figure 2C). Complementation of the *fimA* mutant with the pFimA recombinant plasmid restored its ability to produce the LA/AA-like pattern in the presence of high glucose (Figure 2E). The 1551-2Δ*fimA* strain carrying empty pBADMyc-His A vector (Table 1) produced LA on HeLa cells but was unable to produce the AA-like pattern (Figure 2D), confirming that L-arabinose did not affect the adherence phenotype of the complemented strain.

**Table 1:** **Bacterial strains and plasmids used in this study**

***E. coli*** **strains and plasmids**	**Characteristics**	**Reference**
Strains		
1551-2	aEPEC (ONT:H^−^) isolated from a diarrheic child, which expresses LA on HeLa cells (Nal^R^)	[18]
1551-2Δ*fimA*	1551-2 *fimA*::zeo (Nal^R^ Zeo^R^), T1P mutant	[24]
1551-2Δ*fimA*(pBAD)	1551-2 mutant harboring pBAD/Myc-His A vector (Nal^R^ Zeo^R^ Amp^R^)	[24]
1551-2ΔfimA(p*FimA*)	1551-2 mutant harboring pFimA (Nal^R^ Zeo^R^ Amp^R^)	[24]
Plasmid		
pBAD/Myc-His A	Cloning vector, harboring the araBAD promoter (pBAD) from *E. coli*, which is induced in the presence of L-arabinose.	Invitrogen
pFimA	pBAD/Myc-His A vector harboring the *fimA* gene from aEPEC 1551-2	[24]

Nal^R^: nalidixic acid resistant; Zeo^R^: zeocin resistant; Amp^R^: ampicillin resistant; LA: Localized adherence.

### Presence of bile salts does not interfere with the adhesion properties of 1551–2 strain with HeLa cells

The presence of bile salts caused no significant alterations in the adhesive properties of aEPEC 1551–2 to epithelial cells (data not shown). Under all conditions tested, the adherence pattern and the amount of bacteria associated with HeLa cells were not significantly different, indicating that bile salts did not influence bacterial colonization.

## Discussion

In this study, we analyzed some environmental factors that could influence the colonization process of aEPEC 1551–2 strain in the intestinal environment, by mimicking characteristics (such as the gas atmosphere and glucose concentration) commonly found in different parts of the intestines.

We observed that the efficiency of bacterial association rose approximately two fold in a 5% CO_2_ atmosphere, independently of the glucose concentration employed (Figure 1A). According to described by Babb (1977), hydrogen, nitrogen and CO_2_ are the three prevailing gases present in the intestine, with CO_2_ concentrations varying between 5.1 and 29% [21]. Due to experimental limitations, only 0.03% and 5% CO_2_ were tested.

Haigh and co-workers showed improved protein secretion by the tEPEC E2348/69 strain in the presence of 5% CO_2,_ independently of the presence of eukaryotic cells [22]. However, Kenny and coworkers questioned the actual influence of high CO_2_ concentrations in improving bacterial protein secretion, since, in the adherence assays performed in a 5% CO_2_ atmosphere, sodium bicarbonate is added to the culture medium to buffer the acidic pH induced by the higher CO_2_ concentration [8]. Therefore, the higher protein concentrations observed in such situations could be due to the presence of sodium bicarbonate instead of CO_2_. Since CO_2_ (or sodium bicarbonate) stimulate EPEC protein secretion, we believe that the increased adhesiveness of aEPEC 1551–2 in the atmosphere supplemented with 5% CO_2_ could be related to increased secretion of the EPEC secreted proteins that form the T3SS-translocon (EspA, B and D), in view of the fact that this structure interacts with epithelial cells [23]. In addition, in our previous studies, the T3SS-translocon was shown to mediate aEPEC 1551–2 adherence to HeLa cells in the absence of intimin [24]. Of note, intimin mediates the intimate bacterial attachment observed in the formation of AE lesions [1].

During the flow through the small intestine, glucose is rapidly absorbed, reaching a concentration higher than 25 mM in the duodenum, dropping to less than 5 mM in the ileum, until it is undetectable in the jejunum, depending on the host’s diet [12]. Quantitative experiments to evaluate the efficiency of bacterial interaction with HeLa cells showed no statistical difference between the numbers of associated bacteria when assays were performed in the presence of 5 or 25 mM glucose at the same CO_2_ concentration (Figure 1A).

However, regardless of the CO_2_ concentration used, two distinct adherence patterns were detected depending on the glucose concentration present in the culture medium. At 5 mM glucose, bacteria adhered in the LA pattern, originally reported by Vieira and coworkers [18], whereas at 25 mM glucose, a hybrid pattern consisting of LA and AA-like patterns was seen. Previous studies by our laboratory found that in contact with HeLa cells the 1551–2 strain produces T1P [20]. In addition, this strain lacks adhesin-encoding genes that are commonly found in other diarrheagenic *E. coli* pathotypes and extraintestinal pathogenic *E. coli* (ExPEC), as shown in previous studies [18–20,25].

In the present study, we showed that *fim*A expression increased 1.75-fold and 2.03-fold, in the period between 3 and 6 h, when 25 mM glucose was used as compared to 5 mM glucose. Various studies in the literature have shown that glucose influences the expression of fimbrial and non-fimbrial adhesins. Our results are in accordance with previous studies showing that adhesins such as the bundle-forming pilus (of tEPEC), colonization factor antigens (of enterotoxigenic *E. coli*) and toxin-coregulated pilus (of *Vibrio cholera*) are positively regulated by high glucose concentrations [16,26,27]. Müller and coworkers reported that two uropathogenic *E. coli* strains responded in opposite ways regarding fimbriation in the presence of cAMP receptor protein (CRP) and 3′,5′-cyclic adenosine monophosphate (cAMP) complex by phase variation of *fimA* promoter [28]. CRP-cAMP is a signaling protein complex formed when there is a reduction in glucose concentration in the environment [28]. Therefore, the CRP-cAMP complex can induce the *fimA* promoter in the absence of glucose. However, in the presence of glucose and absence of CRP-cAMP, a complex of Lrp (leucine-responsive protein), DNA gyrase and FimB (an invertase of the T1P operon) raise the amount of fimbriated bacteria in the population by maintaining the phase variation of *fimA* promoter in the ON position, reflecting a glucose–dependent event [28]. In our study, we found that the AA-like pattern of the 1551–2 strain depended on the presence of a high glucose concentration, leading us to hypothesize that the increased *fimA* expression in the later incubation periods corresponded to a glucose-dependent phenotype.

In contrast to the wild-type strain, the isogenic *fimA* mutant lost the ability to produce the AA-*like* pattern, even when the assay was performed in medium supplemented with high glucose concentration, confirming the influence of this sugar in the establishment of the AA-like pattern. The involvement of T1P in the AA pattern was first described in enteroaggregative *E. coli* (EAEC) prototype strain 042, by mutating the *fimD* gene (encoding the T1P usher). The *fimD* mutant strain was approximately 80% less adherent to HEp-2 cells than the wild-type strain [29]. Additionally, T1P has been implicated in biofilm formation in both EAEC (042) and aEPEC (1551–2) strains, reinforcing the involvement of this adhesin in the pathogenesis of diarrheagenic *E. coli* strains [24,29].

As suggested by Edwards and Puente, a combination of environmental signals may provide an intestinal map to identify an appropriate niche for bacterial colonization [12]. The differences in the adherence patterns on HeLa cells under some diverse environmental conditions could reflect the differential expression of adhesin-encoding genes in the distinct parts of the intestine. In view of the evidence that under high glucose concentration the modification in the adherence pattern from LA to LA/AA-like was not associated with an increase of the number of associated bacteria, we can hypothesize that T1P production can negatively interfere with microcolony formation in experiments performed in this circumstance. Therefore, the higher level of T1P production in 25 mM glucose could suggest that these fimbriae are involved in the first steps of the association of aEPEC1551-2 with the proximal small intestine, where the highest glucose concentration is detected [30].

## Conclusions

Altogether, our data suggest that a CO_2_-enriched atmosphere could favor aEPEC adhesion to the host cells, whereas enhanced T1P production under high glucose concentration could allow bacteria to access more extensive intestinal colonization sites in the host at the beginning of the infectious process.

## Methods

### Bacterial strain

The aEPEC 1551–2 strain (serotype ONT:H^−^) was isolated as part of standard patient care from a diarrheic child (23 months old), in the absence of other recognized pathogens, during an epidemiological study of diarrhea carried out at the Universidade Federal de São Paulo (UNIFESP), Brazil. This strain was initially reported to produce LA (in the 6-h assay) in HeLa cells [18]. The *fimA* mutant and complemented strains were obtained in a subsequent study from our laboratory [24]. The most relevant characteristics of the bacterial strains and plasmids employed in the present study are described in Table 1.

Bacterial strains were routinely grown aerobically in Luria-Bertani (LB) medium or Dulbecco’s modified Eagle’s medium (DMEM, Gibco, USA) at 37°C. When appropriate, strains were cultured in the presence of nalidixic acid (20 μg/ml) and/or zeocin (60 μg/ml). Assays employing the complemented strain were performed in the presence of 0.01% L-arabinose as previously described [24].

### HeLa cells culture

HeLa cells were cultivated in DMEM supplemented with 10% fetal bovine serum (Gibco, USA) and in the presence of 1% antibiotic mixture (penicillin and streptomycin, Life Technologies, USA) in an atmosphere of 5% CO_2_ at 37°C. To determine the adherence pattern and quantify the total number of associated bacteria (quantitative assay), HeLa cells were seeded in 24-well microplates, containing glass coverslips and the same media. Adherence assays were performed using semi-confluent cells (approximately 70% confluence) after two days of incubation.

### Qualitative assays (adherence pattern)

Qualitative assays were performed as described earlier [20]. Briefly, HeLa cells were washed with phosphate buffered saline (PBS, pH 7.4). After washing, 1.0 ml of fresh medium (DMEM supplemented with 2% fetal bovine serum) was added to the cell monolayers, and the HeLa cells were then inoculated with suspensions (approximately 10^8^ CFU/mL) of bacteria grown in LB broth (overnight culture) diluted 1:50, and incubated at 37°C. During the interaction, we evaluated the effects of glucose concentration (5 or 25 mM), presence of bile salts (absence or presence of 0.015% w/v) and CO_2_ concentration (0.03 or 5%, with DMEM supplemented with 0.35 or 35 mM sodium bicarbonate, respectively). After 6 h of interaction (with a washing step after 3 h), the preparation was washed six times with PBS and then fixed with methanol (Merck, Germany) for 1 h, stained with May Grünwald-Giemsa stain, and examined by light microscopy for adherence pattern determination.

### Quantitative assays

For determination of the total number of associated bacteria, HeLa cells were inoculated and the experiment was conducted as described above. During the interaction, we evaluated the same assay conditions as in the qualitative assays. After 6 h of interaction (with a washing step after 3 h), the preparations were washed six times with PBS. Next, the cells were lysed with 1% Triton X-100, and serial dilutions were plated on MacConkey agar plates. After approximately 18 h of incubation at 37°C, the resulting colonies were counted for determination of the total number of associated bacteria. All assays were performed in biological and technical triplicate, and the results represented the means ± standard errors.

### Quantitative PCR assay

aEPEC 1551–2 was grown for 18 h in LB broth (or broth supplemented with antibiotics) and incubated statically at 37°C. The pre-inoculum was diluted 1:50 in DMEM supplemented with 5 or 25 mM glucose, and incubated statically for 1.5, 3.0 or 6.0 h, at 37°C, in an atmosphere of 0.03% CO_2_. After incubation, the preparation was centrifuged at 5,900 g for 3 min, and the pellet was treated with Trizol (Invitrogen, USA) for total RNA extraction. cDNA was synthesized using SuperScript III First-Strand (Invitrogen, USA). The primers used for detection of *fim*A expression were: (forward) 5′-TCGATGCGGGATCTGTTGA-3′ and (reverse) 5′-ACCGACGGCAGAGCTGGT-3′. All data were normalized to the expression levels of *rpoA* (RNA polymerase subunit A), by using the following primers: (forward) 5′-GCGCTCATCTTCTTCCGAAT-3′ and (reverse) 5′-CGCGGTCGTGGTTATGTG-3′, and analyzed with the comparative threshold (C_T_) method. The expression levels of *fim*A at different glucose concentrations were compared by using the relative quantification method. Real-time data were expressed as fold change of the expression levels of *fim*A at different glucose concentrations. Data obtained with 25 mM glucose were compared by using the relative quantification method, using strains incubated with 5 mM glucose as the calibrator for each time point. Total RNAs of all treatments were obtained from three independent assays. Statistical differences were determined by the Student *t-*test, and *P* ≤ 0.05 was considered statistically significant.
